# Discrimination between *Mycobacterium tuberculosis* and *Mycobacterium bovis* using Fourier transform infrared spectroscopy

**DOI:** 10.1016/j.onehlt.2026.101356

**Published:** 2026-02-14

**Authors:** Kevim Bordignon Guterres, Taiana Tainá Silva-Pereira, Rodrigo Oliveira, Carolyn G.J. Moonen, Marcos Bryan Heinemann, Flábio Araújo, Moisés Palaci, Gisele Oliveira de Souza, Nathália Silveira Guimarães, Ana Marcia Sá Guimarães

**Affiliations:** a Department of Microbiology, Institute of Biomedical Sciences, University of São Paulo, São Paulo, SP, Brazil; b Bruker Daltonics GmbH & Co. KG, Bremen, Germany; c Department of Preventive Veterinary Medicine, School of Veterinary Medicine, University of São Paulo, São Paulo, SP, Brazil; d Embrapa Beef Cattle, Campo Grande, MS, Brazil; e Department of Pathology, Federal University of Espírito Santo, Vitória, ES, Brazil

**Keywords:** Zoonotic tuberculosis, *Mycobacterium tuberculosis* complex, Machine learning classifier, Diagnostics, IR Biotyper

## Abstract

Zoonotic tuberculosis (TB) caused by *Mycobacterium bovis* (Mbo) is a neglected disease that hinders efforts to eradicate human tuberculosis. Developing a rapid, high-throughput diagnostic test to distinguish Mbo from *Mycobacterium tuberculosis* (Mtb) isolates could enhance global zoonotic TB diagnostics and surveillance. This study aimed to evaluate the ability of Fourier Transform Infrared Spectroscopy (FT-IRS), using the IR Biotyper® system, to differentiate clinical isolates of Mbo and Mtb. Two bacterial inactivation protocols, paraformaldehyde and heat, were tested using Mtb and Bacillus Calmette-Guérin (BCG) strains grown in liquid culture. While both methods allowed FT-IRS analysis, heat inactivation was preferred due to its ease of use and efficiency in biomass recovery. Subsequently, Mtb and Mbo isolates were analyzed using FT-IRS based on polysaccharides, proteins/carbohydrates, and lipids spectral regions, and the resulting spectra were used to construct sample classifiers employing machine learning algorithms. Linear Discriminant Analysis and a UPGMA dendrogram demonstrated clear separations between Mtb and Mbo, particularly for the polysaccharide spectra. Additionally, a classifier built and internally validated using artificial neural networks for the polysaccharide spectra achieved 99% accuracy in distinguishing Mbo and Mtb. Further FT-IRS analysis of few available *Mycobacterium africanum* (Maf) strains demonstrated its capacity to differentiate Maf from Mtb and Mbo, expanding its utility in regions where Maf is endemic. This is the first study to apply FT-IRS to distinguish tuberculous mycobacteria. FT-IRS proved to be a highly effective, rapid, and accurate diagnostic tool for differentiating the Mbo and Mtb strains evaluated in this study, with promising applications for other tuberculous mycobacteria such as Maf.

## 1 Introduction

The *Mycobacterium tuberculosis* complex (MTBC) comprises 11 ecotypes, each adapted to specific hosts and geographic regions [1]. Among these, *M. tuberculosis* (Mtb) is the leading cause of tuberculosis (TB) in humans, accounting for 10.8 million cases and 1.25 million deaths annually [2]. Another notable member, *Mycobacterium bovis* (Mbo), is the main causative agent of zoonotic TB, with an estimated global burden of 147,000 cases and 12,500 deaths per year [3], [4]. Zoonotic TB presents unique challenges to the global control of TB in humans. The disease is clinically undistinguishable from human TB and shows higher proportions of extra-pulmonary cases [5], which complicate diagnosis and delay treatment initiation [4]. Additionally, most strains of Mbo are intrinsically resistant to pyrazinamide [6], a key first-line drug for TB therapy, subjecting diseased individuals to ineffective treatment in the absence of drug resistance testing. Therefore, to end TB in humans, it is crucial to tackle the zoonotic form of the disease.

Major problems in the control of zoonotic TB are its surveillance and diagnostics. The current WHO global TB report does not address zoonotic TB [2] and routine surveillance is absent in 90% of the WHO signatory countries [7]. Current diagnostic tests, such as GeneXpert MTB/RIF Ultra and culture, do not differentiate between Mtb and Mbo. Culturing Mbo is further complicated in Löwenstein-Jensen medium and liquid culture systems, such as BD BACTEC™ MGIT™ 960, due to the absence of pyruvate, which is needed for Mbo growth [8]. Even if Mbo isolates are obtained or new methods are implemented to facilitate their isolation, no rapid tests are available to distinguish Mtb from Mbo, which can only be achieved through molecular biology techniques such as standard, quantitative and multiplex PCR, high-resolution melting (HRM) curve analysis, DNA probes, MIRU-VNTR, spoligotyping, whole-genome sequencing (WGS), or targeted-next generation sequencing (t-NGS) [[6], [9], [10], [11], [12], [13], [14]]. In addition to species identification, MIRU-VNTR and spoligotyping also allow strain-level discrimination, supporting epidemiological analyses [15]. Matrix-assisted laser desorption/ionization time-of-flight mass spectrometry (MALDI-TOF MS), although widely recognized for its speed and effectiveness in microbial identification, lacks the resolution to differentiate species within the MTBC and current commercial platforms do not include validated protocols or reference spectra for distinguishing Mtb from Mbo. Therefore, a rapid, high-throughput test is urgently needed to improve individual diagnostics and surveillance of zoonotic TB. This form of the disease is expected to be more prevalent in countries with a high TB burden, where human cases reach hundreds of thousands. In many of these countries, estimates of zoonotic TB are between 2.2% to 8% of all TB cases [15]. Thus, such test would enable more efficient targeted surveillance of zoonotic TB by simplifying the screening of thousands of MTBC isolates.

Infrared spectroscopy (IR) has found wide-ranging applications in microbiology, with Fourier Transform IR (FT-IRS) providing a fast and cost-effective method for distinguishing closely related microorganisms [16]. FT-IRS is a form of absorption spectroscopy that measures how much infrared light a sample absorbs across a range of wavelengths. Unlike traditional IR spectroscopy, which uses a monochromatic light beam that scans one wavelength at a time, FT-IRS simultaneously exposes the sample to a broad spectrum of infrared frequencies. An interferometer modulates the combined frequencies of the infrared beam by altering the position of a moving mirror, producing an interferogram, which is a time-domain signal that encodes the intensity of absorbed light across all infrared frequencies. Fourier transform is then applied to convert the raw interferogram (i.e. light absorption for each mirror position) into an absorption spectrum, showing absorbance at each specific wavenumber [17]. FT-IRS utility in differentiating biological specimens relies on the principle that similar or closely related samples exhibit comparable absorbance patterns due to their shared molecular composition.

When FT-IR spectroscopy is applied to microorganisms, specific wavenumber regions correspond to major biomolecules, such as polysaccharides (1200–900 cm^−1^), proteins/carbohydrates (1700–1500 cm^−1^), and lipids (3000–2800 cm^−1^), which can be used to differentiate between samples based on their biochemical composition [17]. This approach has been effectively utilized to differentiate between pathogenic and non-pathogenic strains of *Escherichia coli* [18], to distinguish between methicillin-resistant and -sensitive *Staphylococcus aureus* [19], to detect metabolic changes associated with biofilm formation in *Pseudomonas aeruginosa* [20], to identify *Klebsiella pneumoniae, Legionella pneumophila* and *Acinetobacter baumannii* outbreaks [[21], [22], [23]], among others. Importantly, FT-IRS has been successfully employed to differentiate subspecies of *Mycobacterium abscessus* [24] and to trace the epidemiology of *Mycobacterium chimaera* strains in heater-cooler units used in cardiac surgery [25], attesting its use for the *Mycobacterium* genus.

FT-IRS has proven invaluable for high-throughput diagnostic applications in clinical settings. Compared to molecular biology techniques, FT-IRS would offer significant advantages in speed, cost-effectiveness, and ease of integration into clinical workflows to distinguish MTBC species. FT-IRS is less expensive than many molecular biology techniques, making it more accessible in resource-limited settings, and does not require DNA extraction, with sample preparation being fast and easy. Therefore, the aim of this study was to evaluate the IR Biotyper® system (Bruker Daltonics GmbH & Co. KG, Bremen, Germany) for its ability to differentiate between Mtb and Mbo clinical isolates. The developed protocol will enhance the diagnostic accuracy of zoonotic TB, thereby improving the management and control of TB in humans.

## 2 Methods

### 2.1 Mycobacteria strains, culture conditions, and species confirmation

The bacterial strain panel used in this study comprised a combination of clinical isolates and reference strains (Table 1). Accordingly, 18 MTBC isolates were used in this study, including two laboratory strains (Mtb CDC1551 and Mtb H37Rv), one BCG (Bacillus Calmette-Guérin) Pasteur strain 1173 P2, 5 clinical isolates of Mtb, 7 clinical isolates of Mbo, and 3 clinical isolates of *Mycobacterium africanum* (Maf) (Table 1). Some clinical isolates were obtained from established biological resource centers (Belgian Coordinated Collections of Microorganisms, Belgium; or Biodefense and Emerging Infections (BEI) Resources, USA), whereas others were isolated and identified by collaborating laboratories (Table 1). Mbo and Mtb strains were cultured under standardized conditions, and the number of *in vitro* passages prior to analysis was kept to a minimum (≤5 passages) to limit potential phenotypic drift. Exceptions included reference strains (H37Rv, CDC1551, and BCG) and a few isolates for which passage history was unavailable (SV068, M229, and SP38). Detailed strain metadata, including source and lineage are provided in Table 1.

**Table 1:** Isolates of the *Mycobacterium tuberculosis* complex included in this study.

Species	Isolate ID	Sample Origin	Lineage	Reference	Accession number
*M. bovis*	309	Cattle	Eu1	[26]	ERR4450940
	779	Cattle	Eu1		ERR4450948
	B2	Bison	Eu2	[27]	SRR7693912
	Cap	Capybara	Eu2	[[28], [29]]	SRR9850824
	Lh1	Llama	Eu2		SRR7693877
	Lh2	Llama	Eu2		SRR6865435
	SP38	Cattle	Eu2	[30]	SRR6705904
	BCG	–	Pasteur strain 1173 P2	Butantan Institute^A^	–
*M. tuberculosis*	11/21P	Parrot	L4	FMVZ, USP^B^	–
	12/24 A	Tapir	L4	FMVZ, USP	–
	CDC1551	Human	L4	BEI Resources^C^	–
	H37Rv (ATCC 27294)	Human	L4	FMVZ, USP	–
	SVO01	Human	L4	SVOC, FM, USP^D^	–
	SVO68	Human	L4	[31]^E^	–
	M299	Human	L2	[[32], [33]]^F^	–
*M. africanum*	L1222 (M008470)	Human	L6	BCCM^G^	SRR11999301
	NLA000017458	Human	L5	BEI Resources	SRR11999296
	L342 (M003608)	Human	L5	BCCM	–

BCG = Bacillus Calmette-Guérin. L = Lineage (L2, L4, L5 and L6), Eu1 and Eu2 = Clonal Complex European 1 and 2, respectively. ^A.^ Kindly provided by Prof. Dr. Luciana C. de C. Leite from Butantan Institute, Brazil. ^B.^ Bacterial Zoonoses Laboratory from School of Veterinary Medicine of the University of São Paulo (FMVZ, USP). ^C.^ Biodefense and Emerging Infections Research Resources Repository, ATCC, USA. ^D.^ Isolate obtained from the lungs of an autopsied patient from the “Serviço de Verificação de Óbito da Capital” (SVOC), School of Medicine, USP. ^E.^ Núcleo de Doenças Infecciosas, Federal University of Espirito Santo (NDI, UFES) ^F.^ Kindly provided by Prof. Dr. Elena B. Lassonskaia from Universidade Estadual of Norte Fluminense Darcy Ribeiro (UENF). ^G.^ Belgian Coordinated Collections of Microorganisms, Belgium.

The bacteria were maintained in Löwenstein-Jensen, Stonebrink or Middlebrook 7H9 (BD, USA) supplemented with 10% OADC [oleic acid (Synth, Brazil), albumin (Invitrogen, USA), dextrose, sodium chloride, and catalase (Sigma-Aldrich, USA)], 18 mM sodium pyruvate (Dinâmica, Brazil), and 0.05% Tween 80 (Inlab, Brazil) (7H9-OADC-Pyr-Tween). Isolates were stored at −80 °C in 7H9-OADC-Pyr-Tween with 20% glycerol.

To confirm the species of each strain, DNA of the bacteria grown in 7H9-OADC-Pyr-Tween were extracted using a previously described protocol [34] and subjected to a multiplex-PCR to identify RDs (regions of difference) as described [11]. RD-based multiplex PCR was also performed before and after experiments to control cross-species contamination. In addition, the genomes of all Mbo and two Maf isolates have been sequenced by our group or others and published previously (Table 1), which further supports their species and clonal complex/lineage identification.

All procedures involving live mycobacteria were performed in a Biosafety Level 3+ Laboratory (BSL3+ Prof. Dr. Klaus Eberhard Stewien), located in the Department of Microbiology, Institute of Biomedical Sciences (ICB), University of São Paulo (USP), Brazil.

### 2.2 Culture and inactivation protocols

Considering the biosafety requirements to work with tuberculous mycobacteria, an alternative inactivation method to the IR Biotyper kit protocol was sought. For these, a clinical isolate of Mtb (strain SVO01) and a BCG strain were evaluated with different protocols and compared to the kit inactivation protocol using the BCG strain only, which is a BSL2 (biosafety level 2) bacterium. Two protocols were tested: paraformaldehyde (PFA) and heat inactivation. These are commonly used methods of mycobacteria inactivation [35]. Briefly, −80 °C stocks of MTBC strains were thawed, washed twice with fresh 7H9-OADC-Pyr-Tween, inoculated onto the same media and incubated at 37 °C for 5 days in T25 flasks (Greiner Bio-One). Subsequently, 1 mL aliquots were transferred to T75 flasks (Greiner Bio-One) containing 9 mL of 7H9-OADC-Pyr-Tween and cultured for 6–7 days at 37 °C, until an optical density at 600 nm (O.D._600_) of 0.6–0.8. Ten mL of the cultures were centrifuged at 2000 x*g* for 15 min, resuspended in 1 mL of sterile distilled water, and passed through a 26-gauge needle five times to eliminate bacterial clumps. The suspension was then inactivated using PFA, heating or the kit solution (which is 70% ethanol). The PFA protocol consisted in adding the solvent to the suspension to a concentration of 4% and incubating for 40 min at 4 °C covered from light. The suspension was then centrifuged at 2000 x*g* for 15 min, washed twice with sterile distilled water, and resuspended in a mixture of 50 μL of 70% ethanol and 50 μL of sterile distilled water in an IR Biotyper® suspension vial (Bruker Daltonics GmbH & Co. KG). The heat inactivation method consisted of subjecting the suspension to a dry-block incubation at 95 °C for 30 min. The suspension was then centrifuged at 2000 x*g* for 15 min and resuspended in a mixture of 50 μL of 70% ethanol and 50 μL of sterile distilled water in the suspension vial. The suspension containing the BCG strain was subjected to the manufacturer's inactivation protocol, according to instructions, which consists of ethanol inactivation. Experiments were conducted using 5 replicates of each strain.

Prior to IR Biotyper analysis, aliquots derived after PFA and heat inactivation were inoculated into rich medium (7H9-OADC-Pyr-Tw and 7H10-OADC-Pyr) and incubated at 37 °C for 6 weeks to test the efficacy of inactivation, showing no growth.

### 2.3 FT-IRS spectra acquisition and data analysis

For the FT-IRS analysis, 24 mL of culture from each strain was used following the same protocol described above. The isolates were analyzed in four to five technical replicates over three independent experiments, whereas the Mtb H37Rv strain was evaluated in six independent experiments. A 15 μL aliquot of each bacterial suspension was spotted onto a 96-well silicon IR Biotyper® plate (Bruker Daltonics GmbH & Co. KG) and dried at 37 °C for 15–20 min, according to the manufacturer's protocol. Additionally, 10 μL each of two IR Test Standards (IRTS1 and IRTS2) were spotted in duplicates.

Spectra were acquired (transmission mode between wave numbers 4000–500 cm^−1^) and processed by OPUS software *V*.8.2.28 (Bruker Optics, Ettlingen, Germany) on an IR Biotyper® with the corresponding IR Biotyper® software V4.0 (Bruker Daltonics GmbH & Co. KG) for data analysis. After measurement, each spectrum was subjected to a Quality Test (QT). Parameters such as absorption, noise (x 10^6^), signal-to-noise ratios (R2 and R3), signal-to-water ratios (R2 and R3), and fringes (x 10^6^) were analyzed for each spectrum. As samples were run in quadruplicates or quintuplicates, four or five spectra were obtained per sample. If all five spectra failed quality control (i.e., QT fail with a yellow warning), the entire sample was excluded from the analysis and reprocessed using a new culture batch. If fewer than five spectra failed, individual spectra were excluded if they met at least one of the following criteria: (i) signal-to-water ratio below 200 in region R2 or below 40 in region R3, or (ii) QT fail with a yellow warning and visible divergence from the other replicates of the same sample in the LDA plot (i.e., failure to cluster). A minimum of three valid replicates per sample was required for inclusion in the analysis. Spectral splicing was performed using the default polysaccharide region of 1300–800 cm^−1^ to evaluate mycobacterial inactivation protocols. When analyzing the mycobacterial isolates, all three regions (polysaccharides region of 1300–800 cm^−1^, proteins/carbohydrates region of 1700–900 cm^−1^ and lipids region of 3000–2800 cm^−1^) were analyzed and compared.

Spectral distances were visualized using dimension reduction tools integrated into the IR Biotyper® software. Accordingly, Principal Component Analysis (PCA) or Linear Discriminant Analysis (LDA) with isolate ID set as the target group was used to generate 2D and 3D plots capturing up to 100% of the variance using a maximum of 30 principal components. Isolate ID was used as the target group in LDA, instead of species, to avoid overfitting or a false group separation. To visualize differences across all principal components, deviation plots were created using isolate ID and species as target groups, where the solid line represents the mean spectrum, and the shaded area indicates the standard deviation.

Dendrograms using UPGMA algorithm with correlation distance were constructed to demonstrate the interrelation proximity of the samples at a certain distance. Each dendrogram displays a horizontal axis of the spectral distance, reflecting dissimilarity between FT-IRS spectra of the isolates, with lower values indicating higher similarity. The software automatically calculated a cutoff of this spectral distance based on SDI (Spectral Distance Index) x mC (mean Concordance), which is displayed as a vertical line crossing the branches of the dendrogram. Isolates associated with branches merging at spectral distance values below the cutoff are considered to belong to the same cluster, while those with branches merging at higher values are classified into distinct clusters, suggesting greater spectral dissimilarity and potentially indicating different mycobacterial species.

### 2.4 Sample classifier

FT-IRS classifiers enable automated classification of unknown spectra using machine learning algorithms. These classifiers combine a machine learning model, such as an artificial neural network (ANN) or Support Vector Machine (SVM), and an outlier detector (OD). For each spectral region, a classifier was developed from labeled spectra, where a set of 242 spectra, representing 7 Mbo and 7 Mtb isolates, was used to define two groups. The machine learning models, implemented as a stepwise approach within the software, learn to recognize the specific features of the labeled spectra, classifying unknown samples as either Mtb or Mbo based on these patterns. The OD further evaluates how closely the samples match the training set, assigning a reliability score using a “traffic light” system: green for high confidence, yellow for moderate confidence, and red for low reliability, possibly indicating an unknown or significantly different isolate. Accordingly, for each spectral region, the dataset of 242 spectra was divided into two groups: a training dataset (total of 124 spectra) consisting of 4 Mtb and 3 Mbo isolates and a testing dataset with 3 Mtb and 4 Mbo isolates (total of 118 spectra) (Table S1). The training dataset of 124 spectra was used to create a reduced classifier for each spectral region using the same machine learning algorithm (ANN, 500 cycles). Next, these classifiers were applied to the “unknown” testing dataset of 118 spectra resulting in confusion matrices displaying the accuracy of that reduced classifiers. The purpose of the classifier trained on a subset of spectra (referred as “reduced classifier”) was to evaluate the model's ability to generalize to unseen data. By training on a portion of the dataset (124 spectra) and testing on a separate subset (118 spectra), we aimed to simulate real-world diagnostic scenarios and assess the classifier's accuracy.

## 3 Results

### 3.1 Evaluation of mycobacterial inactivation protocols

**Fig. 1:**
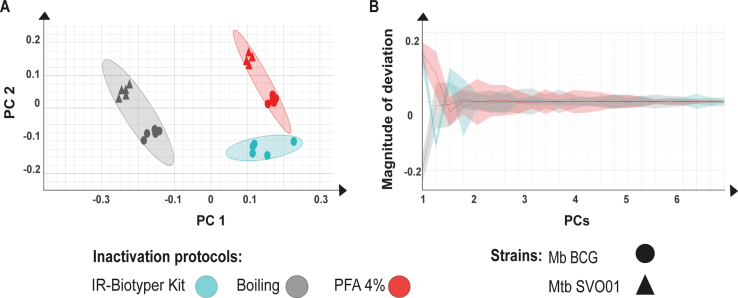
PCA (Principal Component Analysis) and deviation plot of and *Mycobacterium bovis* BCG Pasteur and *Mycobacterium tuberculosis* SVO01 inactivated with different protocols and subjected to Fourier Transform Infrared Spectroscopy. A) Two dimensions PCA (69.33% of the data variance). Inactivation methods are depicted with different colors, according to the legend. B) Spectra deviation plot for BCG (polysaccharide region of 1300–800 cm^−1^). Solid line indicates the mean spectrum, and shaded area represents the standard deviation.

PCA results of Mtb SVO01 and BCG samples subjected to different inactivation protocols are shown in Fig. 1. Accordingly, Mtb SVO01 and BCG samples cluster separately, but more closely according to the inactivation method used (PFA or heating) (Fig. 1). The PCA graph also shows that the cluster of BCG samples inactivated with the kit protocol appeared in an intermediate position between the samples inactivated with PFA and heating (Fig. 1). Therefore, while different inactivation methods cluster separately, there was evidence of mycobacterial species distinction for both PFA and heat inactivation protocols.

All samples met the analyzed QT parameters, except for the absorption values. The absorption values were below the reference range of 0.4–2 in all samples (mean: 0.2, std.: 0.7), except for two samples of BCG subjected to the heat inactivation method. This suggests that the biomass obtained from 10 mL of cultured media was insufficient to achieve absorption values within the reference range, even though the sample replicates clustered well in the PCA. For this reason, the next experiments were conducted with 24 mL of cultured media and using the heat inactivation method. Heat inactivation was chosen because it separated Mtb from BCG in a similar manner to PFA, is easier to be applied in different laboratories, and requires fewer washes compared to PFA inactivation, resulting in greater amount of final biomass.

### 3.2 FT-IRS discriminates between Mtb and Mbo strains

A total of 264 spectra were generated across all FT-IRS runs performed in this study. These include repetitions of two samples that initially failed quality control in one run, with all five spectra presenting QT fail with yellow warning, and were therefore re-cultured, reprocessed, and re-analyzed. Of the remaining 254 spectra, nine were excluded due to low signal-to-water ratios, and three were excluded because they showed a QT fail with yellow warning and did not cluster with their corresponding replicates on the LDA plot. Therefore, a total of 242 spectra were included in the final analysis, with all included samples satisfying the requirement of at least three valid replicates per run and three independent experiments.

**Fig. 2:**
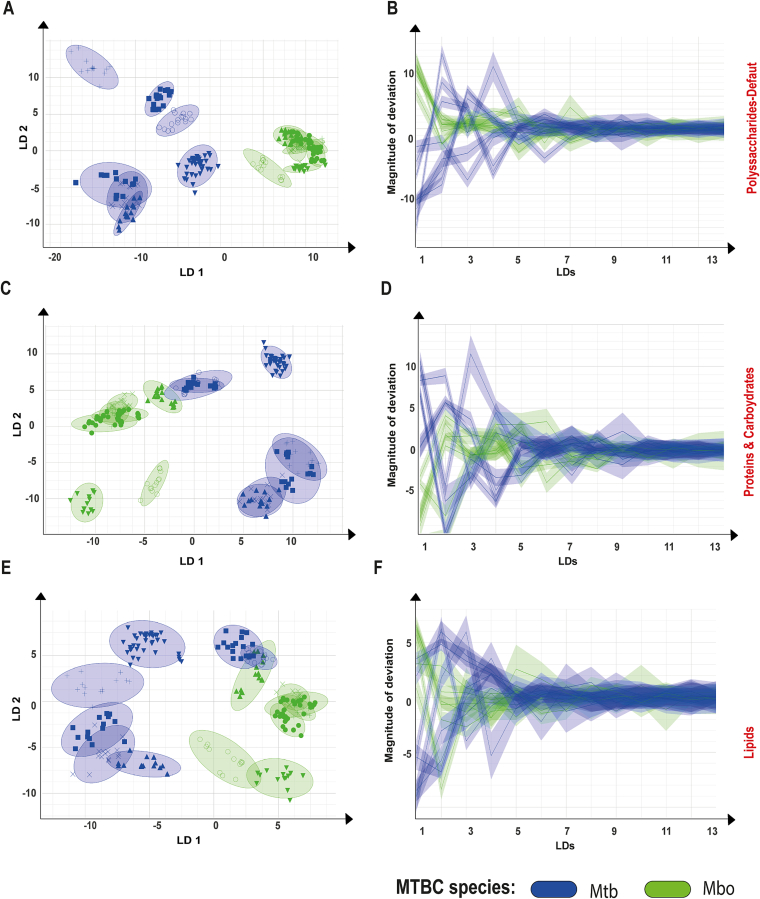
Linear Discriminant Analysis (LDA) and deviation plots of isolates of *Mycobacterium tuberculosis* (Mtb, blue) and *Mycobacterium bovis* (Mbo, green) subjected to Fourier Transform Infrared Spectroscopy. In the LDA plots, isolates are represented by different shapes and are encircled to indicate their replicates. (A) 2D scatter plot of polysaccharide-based spectra of Mtb and Mbo strains (LDA: 30 LDs, 99.1% variance, target group = isolate ID). The x-axis shows LD1 (59.00%) and the y-axis shows LD2 (15.78%), together displaying 74.78% of the variance. (B) Deviation plot of polysaccharide-based spectra of Mtb and Mbo strains. The solid line indicates the mean spectrum, and the shaded area represents the standard deviation. The x-axis displays different linear components. (C) 2D scatter plot of protein /carbohydrate-based spectra of Mtb and Mbo strains (LDA: 30 LDs, 99.7% variance, target group = isolate ID). The x-axis shows LD1 (41.29%) and the y-axis shows LD2 (32.66%), together displaying 73.95% of the variance. (D) Deviation plot of protein/carbohydrate-based spectra of Mtb and Mbo strains. The solid line indicates the mean spectrum, and the shaded area represents the standard deviation. The x-axis displays different principal components. (E) 2D scatter plot of lipid-based spectra of Mtb and Mbo strains (LDA: 30 LDs, 100% variance, target group = isolate ID). The x-axis shows LD1 (48.13%) and the y-axis shows LD2 (31.58%), together displaying 79.71% of the variance. (F) Deviation plot of lipid-based spectra of Mtb and Mbo strains. The solid line indicates the mean spectrum, and the shaded area represents the standard deviation. The x-axis displays different principal components. (For interpretation of the references to colour in this figure legend, the reader is referred to the web version of this article.)

Two-dimensional (D) and 3D LDA plots of Mtb and Mbo strains based on the polysaccharide, protein/carbohydrate, and lipid spectral regions are shown in Fig. 2 and fig. S1, respectively. Clear separations between the Mbo and Mtb strains are observed in LDA plots derived from polysaccharide and protein/carbohydrate spectral regions (Figs. 2A, C and Fig. S1). A small overlap between Mtb CDC1551, Mtb SVO01, and Mbo 779 is observed in the LDA plot from the protein/carbohydrate region, while an even more significant overlap among these strains occurred in the lipid-based plot (Fig. 2C and E). In all three LDA plots, most of the variance that distinguishes Mtb and Mbo is captured by the first two to four linear discriminants (LD1–LD4), with LD1 and LD2 accounting for the majority of class-separating variance (Fig. 2).

Deviation plots using “species” as the target group (Fig. S2) show consistently large standard deviations among Mtb strains across all three spectral regions: polysaccharides, proteins/carbohydrates, and lipids. In contrast, Mbo strains exhibited smaller standard deviations in the polysaccharide-based deviation plot compared to the protein/carbohydrate and lipid regions (Fig. S2). These findings likely reflect the greater intra-species variability among Mtb strains compared to Mbo strains, particularly as seen in the polysaccharide-based LDA plot (Fig. 2A, C, E). Taken together, these findings reinforce the value of FT-IRS, particularly using the polysaccharide region, to distinguish between the mycobacterial species.

**Fig. 3:**
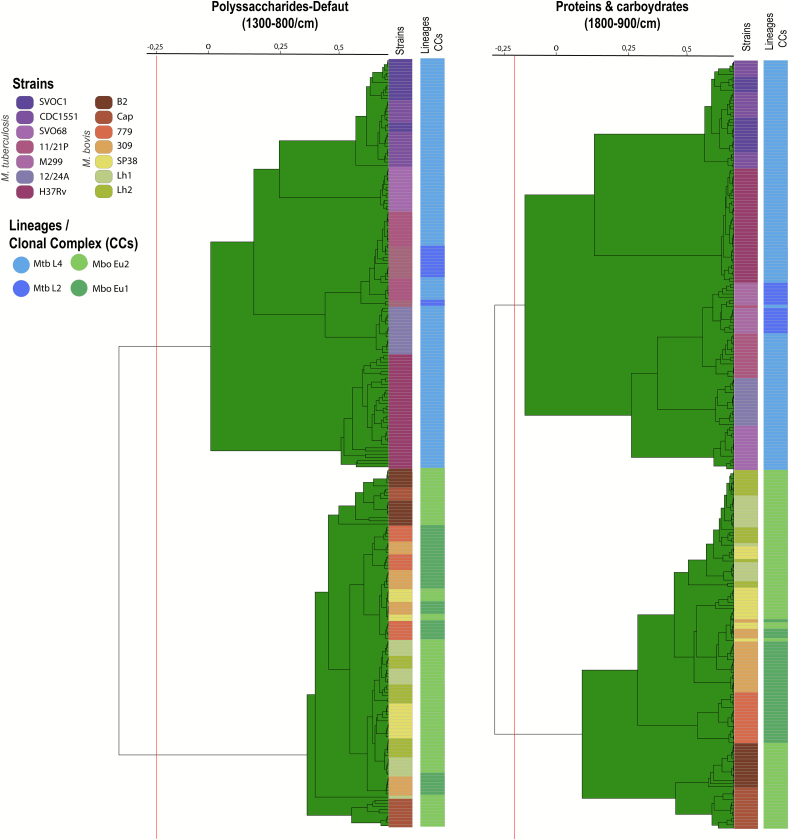
Dendrograms based on polysaccharide and proteins/carbohydrates spectral regions of *Mycobacterium tuberculosis* (Mtb) and *Mycobacterium bovis* (Mbo) isolates analyzed by Fourier Transform Infrared Spectroscopy (FT-IRS). Spectral splicing was performed using the polysaccharide range (1300–800 cm^−1^) and the protein/carbohydrate range (1700–900 cm^−1^). The UPGMA algorithm with correlation distance was applied. The horizontal axis represents spectral distance, indicating differences between FT-IRS spectra of the isolates. Spectral distance cutoffs, automatically determined by the software using the Spectral Distance Index (SDI) and mean Concordance (mC), are shown as vertical lines intersecting the dendrogram branches. These cutoffs highlight two distinct and homogeneous clades in each dendrogram, corresponding to Mtb and Mbo strains. Green-colored branches indicate homogeneous clusters composed of strains from the same species; otherwise, the software would display them in yellow. (For interpretation of the references to colour in this figure legend, the reader is referred to the web version of this article.)

Dendrograms derived from polysaccharide and protein/carbohydrate spectral regions distinctly separate Mtb and Mbo strains into two well-supported clades, as indicated by the branching patterns and the spectral distance cutoff automatically determined by the software (Fig. 3). In contrast, the dendrogram based on the lipid spectral region did not resolve Mtb and Mbo strains into separate clades (Fig. S3). Consistent with the lipid-based LDA results (Fig. 2E), strains Mbo 779, Mtb SVO01, and CDC1551 clustered together, preventing clear discrimination between Mtb and Mbo in this spectral region (Fig. S3). Finally, while the number of strains from each clonal complex of Mbo is low (two Eu1 and four Eu2), we did not observe a clear discrimination between them in any of the dendrograms (Fig. 3 and S3).

### 3.3 A classifier for Mtb and Mbo

**Fig. 4:**
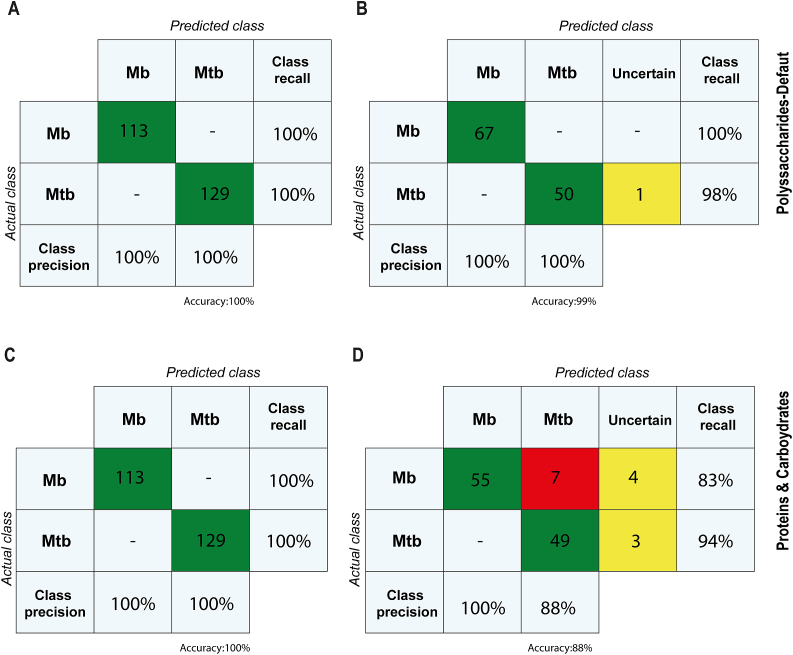
Confusion matrices of classifiers generated with spectra from *Mycobacterium tuberculosis* and *Mycobacterium bovis* isolates subjected to Fourier Transform Infrared Spectroscopy. A) Confusion matrix as output from classifier creation of 242 spectra of polysaccharides spectral region in the IR Biotyper® software. Vertical axis displays the actual class as labels (input given by user), and the horizontal axis displays the predicted classes by the classifier model. The numbers in green depict the number of spectra. B) Confusion matrix as output from testing the reduced classifier (made with 124 spectra of polysaccharides spectral region) tested on 118 “unknown” spectra of polysaccharides spectral region in the IR Biotyper® software. The numbers in green depict the number of spectra (67 and 50 for Mbo and Mtb, respectively) predicted correctly (matching the actual class), and yellow depict the number of spectra which are uncertain. C) Confusion matrix as output from classifier creation of 242 spectra of proteins/carbohydrates spectral region in the IR Biotyper® software. Vertical axis displays the actual class as labels (input given by user), and the horizontal axis displays the predicted classes by the classifier model. The numbers in green depict the number of spectra. D) Confusion matrix as output from testing the reduced classifier (made with 124 spectra of polysaccharides spectral region) tested on 118 “unknown” spectra of polysaccharides spectral region in the IR Biotyper® software. The numbers in green depict the number of spectra (55 and 49 for Mbo and Mtb, respectively) predicted correctly (matching the actual class), yellow depict the number of spectra which are uncertain and red the number of spectra which are incorrect. (For interpretation of the references to colour in this figure legend, the reader is referred to the web version of this article.)

The spectral distance displayed by the Mtb and Mbo isolates can be learned by a machine learning algorithm for future automated classification of unknown spectra by the IR Biotyper® software. Since the LDA and dendrogram analysis of lipid spectral region was not able to separate Mtb from Mbo, we created classifiers based on polysaccharide and protein/carbohydrate spectral regions only. These classifiers were created with the three available machine learning algorithms using a stepwise approach integrated in the IR Biotyper® software. Initially, Support Vector Machine (SVM) with a Radial Basis Function (RBF) and SVM with a linear kernel were explored as machine learning algorithms for both spectral regions but did not reach >95% accuracy, hence these machine learning algorithms were not further used. Classifiers were then created with a total of 242 spectra using the machine learning algorithm Artificial Neural Networks, reaching 100% accuracy for both spectral regions (ANN, 500 cycles, Fig. 4A and C). Subsequently, reduced classifiers with a total of spectra were created (100% accuracy, confusion matrix not shown) and validated with other 118 spectra of the testing isolates (Fig. 4B and D).

Fig. 4B shows the polysaccharides-based confusion matrix of the testing dataset of 118 spectra after applying the “reduced” classifier, created with 124 spectra. There are two actual classes (labeled isolates) and the output of the predicted classes by the newly created classifier (Fig. 4). All spectra, except for one spectrum of isolate Mtb M299, was predicted correctly (Fig. 4B). Of the isolate M299, the prediction was 93.3% Mtb, where one spectrum only was predicted as second-best result Mbo. Overall, this resulted in the internal validation of this classification model as 99% accurate.

Fig. 4D shows the proteins/carbohydrate-based confusion matrix of the testing dataset of 118 spectra after applying the “reduced” classifier, created with 124 spectra. Seven spectra were predicted incorrectly and other seven were uncertain, resulting in lower accuracy compared to the polysaccharide-based model. Taken together, these results indicate that the generated classifier based on polysaccharide spectral region, created with the total dataset of 242 spectra, would be better suited for species classification compared to the proteins/carbohydrates-based model, and is ready to be used for further testing with new isolates as external validation.

### 3.4 The potential use of FT-IRS to distinguish among additional MTBC species

**Fig. 5:**
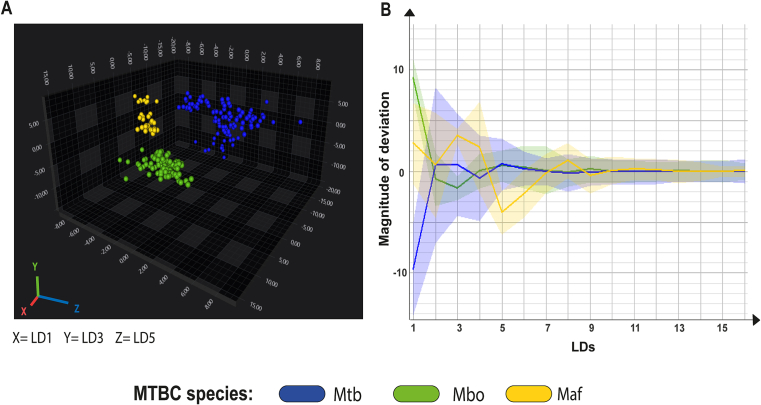
Linear Discriminant Analysis (LDA) and deviation plot of *Mycobacterium tuberculosis* (Mtb), *Mycobacterium bovis* (Mbo) and *Mycobacterium africanum* (Maf) isolates subjected to Fourier Transform Infrared Spectroscopy. A) 3D scatter plot (LDA 30 LDs, 99.3% variance, target group = isolate ID) showing Mtb isolates in blue, Maf in yellow and Mbo in green. The x-axis shows LD1(54.97%) y-axis shows LD3 (8.45%), and z-axis shows LD5 (3.61%) together displaying 67,03% of the variance. B) Deviation plot displaying Mtb, Maf and Mbo isolates. Solid line indicates the mean spectrum, and shaded area represents the standard deviation. X axis displays different principal components. (For interpretation of the references to colour in this figure legend, the reader is referred to the web version of this article.)

Maf is another MTBC species of great importance for human health. However, this species is endemic in West Africa and not in Brazil, where this study was conducted. For this reason, we did not have many isolates of Maf to be evaluated. Only three isolates, acquired through importation from reference databases of microorganisms, were available. Nevertheless, we opted to subject them to FT-IRS analysis and compare them to Mbo and Mtb spectra. Fig. 5A illustrates the polysaccharide-based LDA distribution profiles for the three species using LD1, LD3, and LD5 regions. It reveals three isolated clusters, highlighting the technique's potential to address the challenge of differentiating other species. One of the L5 strains exhibited a distinct profile compared to other Maf strains in the initial LD regions (Fig. S4), but this difference was mitigated when focusing on a region that shared greater similarity with the species, as observed in LD5 (Fig. 5B). Unfortunately, LDAs based on proteins/carbohydrates and lipids spectra were unable to differentiate among the three species (Fig. S5), suggesting that the polysaccharide spectral region is the most appropriate choice for distinguishing species within the MTBC.

## 4 Discussion

This study presents an innovative diagnostic method for differentiating between Mtb and Mbo, with potential applicability to other species within the MTBC. The method offers a rapid, high-throughput test to support diagnostics and surveillance of zoonotic TB and Maf infections. In addition, we showed that the FT-IRS analysis using the IR Biotyper® can be performed on MTBC bacteria cultured in liquid media, which was not the standard protocol [[16], [24]]. The conventional approach required processing samples from solid media. This finding is significant because routine TB diagnostics often rely on automated liquid culture systems, such as the BD BACTEC™ MGIT™ 960, enabling the rapid identification of isolates. Integrating automated liquid media systems with subsequent FT-IRS analysis would enhance uniformity across laboratories, improve quality control, and reduce errors [[2], [36]].

Due to the alcohol-acid resistance of mycobacteria, an inactivation method other than the standard 70% ethanol recommended by the manufacturer was required. Our results demonstrate that the methods tested (PFA and heating) exhibit distinct clustering behaviors in PCA. This is expected, as heating induces cell lysis through heat, while PFA likely preserves the cell wall structure by fixing the bacteria [37]. Previous studies have reported that sample preparation and prior exposure to adverse conditions can affect FT-IRS spectra [[38], [39]]. In this study, our objective was not to preserve cellular ultrastructure or obtain spectra of specific unaltered biomolecules, but rather to accurately differentiate between ecotypes of the MTBC based on their overall biochemical fingerprint. For diagnostic purposes, heat inactivation was selected as an efficient and widely applicable method that can be easily implemented in laboratories worldwide. In this context, potential alterations to specific molecular features are less critical, provided that the resulting spectra are consistent and reproducible for distinguishing Mtb from Mbo. Our results demonstrate that, despite any spectral shifts due to inactivation, classification performance remains extremely high (99% accuracy), indicating the method's robustness under the tested experimental conditions. Importantly, our results indicate that consistent and homogeneous sample preparation should be used when applying FT-IRS.

The default spectral wavenumber range of polysaccharide yielded the best results for species differentiation using the strain panel described herein. Given the critical role of lipids in MTBC physiology and known interspecies differences in cell wall composition, we anticipated species-specific distinctions in the lipid region of the spectrum; however, these were not observed. On the other hand, the limited resolution in the protein-associated spectra was expected, due to the high genomic similarity between ecotypes. Nevertheless, it would be interesting to test if, by maintaining the cells intact with PFA, further differentiation can be achieved among lineages and clonal complexes of each species based on the lipids spectra. This is particularly significant because mycobacteria possess a lipid-rich cell wall, with many glycolipids, that are markedly different from those of gram-positive and gram-negative bacteria [40]. Thus, this technique holds potential for further applications and exploration.

An interesting result of our study is the larger standard deviation observed in the group spectra of Mtb compared to Mbo. Mtb isolates also appeared more dispersed in LDA graphs compared to Mbo, particularly in the polysaccharide spectral region. These findings suggest that the cell wall of Mtb is more diverse than Mbo, with greater variation in polysaccharides (e.g. arabinogalactan and capsular polysaccharides) and/or molecules containing polysaccharides, such as lipoarabinomannan (LAM), trehalose dimycolate (TDM), and phosphatidyl-myo-inositol mannosides (PIM). Interestingly, this greater variability of Mtb spectra aligns with the fact that Mtb strains are more genetically diverse than Mbo strains. Despite being clonal and having highly similar genomes, the mean pairwise SNP-distances between Mtb genomes are significantly higher than those of Mbo strains [[41], [42]], even when only Mtb L4 strains are considered [43]. This is likely because Mbo is a more recently evolved member of the MTBC [[44], [45]]. However, since only seven isolates of each species were analyzed, these findings should be further confirmed using a broader panel of Mtb and Mbo isolates from diverse global regions.

Mtb H37Rv, the most used reference strain in TB research, was found to cluster separately from other Mtb isolates in the polysaccharide-based dendrogram, suggesting that it may not be an appropriate standard to be used in FT-IRS of the MTBC. Isolated in the 1905 [46], Mtb H37Rv has been distributed to laboratories worldwide and has undergone an unknown number of culture passages. These factors have led to a reduction in virulence [[47], [48]] and other potentially unknown changes to its phenotype, which may explain our findings.

A sample classifier based on the polysaccharide spectral region for distinguishing Mtb and Mbo was successfully developed to identify unknown spectra. One spectrum only, from isolate Mtb M299, was misclassified as Mbo. However, this isolate would still be assigned as Mtb with over 90% certainty because the other spectra were assigned to the right class. Interestingly, Mtb M299 was the only Mtb L2 used in this work. Due to the higher variability in Mtb spectra, future studies should include more Mtb and Mbo isolates to enhance the classifier's accuracy to 100%.

Maf was included in this study due to its significance as a species infecting humans in West Africa [49]. Our results are promising for the potential of FT-IRS in distinguishing this species, demonstrating that this technique can effectively differentiate between Mbo and Maf with a level of resolution that even MALDI-TOF (Matrix Assisted Laser Desorption/Ionization – Time of Flight) struggle to achieve [50]. This capability underscores FT-IRS's superior sensitivity to subtle biochemical differences, particularly in the polysaccharide-rich regions of the bacterial cell wall, which seem to be crucial for identifying closely related MTBC species. Moreover, FT-IRS offers a significant advantage for clinical applications over MALDI-TOF, as it eliminates the need for additional extraction steps [50]. Unfortunately, because of the low number of isolates, the sample classifier was not built for Maf.

It is important to contextualize FT-IRS within the existing diagnostic landscape. Several molecular methods are available for differentiating Mtb and Mbo with high discriminatory power; however, many require specialized infrastructure, trained personnel, extended turnaround times, or complex data interpretation, which can limit their routine implementation, particularly in resource-constrained settings. Importantly, IR Biotyper® is neither portable nor a point-of-care strategy, as it requires dedicated instrument and appropriate laboratory infrastructure, as well as prior culture and isolation of the organism, which remains a major time-limiting step. However, within laboratories that already possess minimal microbiological infrastructure, FT-IRS offers a relatively rapid downstream workflow once isolates are available, with short acquisition times, limited hands-on steps, and automated spectral analysis. Therefore, its potential utility lies in reference or clinical laboratories where cultures are routinely obtained and processed. Additional limitations include the need for standardized culture conditions and access to curated reference spectral databases. Overall, FT-IRS should be viewed as a complementary laboratory tool rather than a standalone diagnostic solution. It can support high-throughput screening and species differentiation at a relatively low per-sample cost [[22], [51]], despite the initial investment in instrumentation, particularly given its applicability to multiple bacterial pathogens and rapid analysis time.

Limitations of this study include the lower number of strains, especially for Maf, and the lack of other MTBC species. Nevertheless, results show that differentiation of Mtb and Mbo can be achieved. Furthermore, the created classifier needs external validation. Only an internal validation was done. Ideally, more isolates should be used. However, the created classifier can be easily re-trained with new isolates using the IR Biotyper® software in the future. One limitation to the real-world application of this technique is the amount of biomass needed, and the time required to acquire it. In this study, we adopted a pragmatic approach by using liquid culture media, as it remains the most widely employed in routine TB diagnostics. However, further investigation is warranted to compare alternative biomass acquisition strategies, including extended primary cultivation in MGIT960, biomass amplification via subcultivation in MGIT960, or primary/subcultivation using Löwenstein-Jensen or Stonebrink solid media. Few individual spectra failed QT (i.e. had a yellow warning) due to absorption issues, which is directly associated with the amount of biomass in the sample. The final bacterial suspension transferred to the silicon IR-Biotyper plate was often thick and difficult to homogenize. Despite the use of beads and vortexing between each replicate loading, the last replicate was frequently the one that failed the absorption parameter.

In conclusion, FT-IRS analysis of polysaccharide-based spectra effectively differentiated between the Mtb and Mbo strains evaluated in this study. The sample classifier developed in this study represents a valuable tool for incorporating this technique into routine diagnostics and surveillance of the MTBC. FT-IRS also shows significant potential for distinguishing other species within the MTBC, potentially offering greater resolution than MALDI-TOF. This advantage likely results from the fact that polysaccharides and lipids provide stronger markers for species differentiation in this clonal group compared to the protein-based approach of MALDI-TOF.

## Declaration of generative ai and ai-assisted technologies in the manuscript preparation process

During the preparation of this work the authors used ChatGPT in order to review proper English grammar and sentence structure for parts of the text. After using this tool/service, the authors reviewed and edited the content as needed and take full responsibility for the content of the published article.

## CRediT authorship contribution statement

**Kevim Bordignon Guterres:** Writing – review & editing, Writing – original draft, Visualization, Validation, Methodology, Investigation, Formal analysis, Data curation, Conceptualization. **Taiana Tainá Silva-Pereira:** Writing – review & editing, Formal analysis, Data curation, Conceptualization. **Rodrigo Oliveira:** Writing – review & editing, Writing – original draft, Validation, Supervision, Software, Resources, Project administration, Formal analysis, Data curation, Conceptualization. **Carolyn G.J. Moonen:** Writing – review & editing, Writing – original draft, Supervision, Software, Conceptualization, Validation, Resources, Formal analysis, Data curation. **Marcos Bryan Heinemann:** Writing – review & editing, Visualization, Validation, Resources, Project administration, Formal analysis, Conceptualization. **Flábio Araújo:** Writing – review & editing, Visualization, Validation, Resources. **Moisés Palaci:** Writing – review & editing, Visualization, Validation, Resources. **Gisele Oliveira de Souza:** Writing – review & editing, Visualization, Validation, Resources, Methodology. **Nathália Silveira Guimarães:** Writing – review & editing, Visualization, Validation, Methodology, Resources. **Ana Marcia Sá Guimarães:** Writing – review & editing, Writing – original draft, Visualization, Validation, Supervision, Resources, Project administration, Methodology, Investigation, Funding acquisition, Formal analysis, Data curation, Conceptualization.

## Funding

This study was funded by São Paulo Research Foundation (FAPESP), grant 2023/13388–9. K.B.G.'s fellowship is funded by FAPESP grant 2023/07582–7. T.T.S.P.'s fellowship was funded by Coordination for the Improvement of Higher Education Personnel (CAPES, grant 88887.508739/2020–00). A.M.S.G and M.B.H. are fellows of the National Council for Scientific and Technological Development (CNPq, grants 311543/2023–5 and 309146/2017). CAPES (grant 001) provided graduate studies support.

## Declaration of competing interest

R.O. and C.G.J.M. are employees of Bruker Daltonics GmbH and Co. KG, Germany. The remaining authors declare that the research was conducted in the absence of any commercial or financial relationships that could be construed as a potential conflict of interest.

## Data Availability

Data will be made available on request.

## Appendix A Supplementary data

Supplementary material Image 1
