# Supplementary material for: Discrimination between Mycobacterium tuberculosis and Mycobacterium bovis using Fourier transform infrared spectroscopy
Source: One Health. 2026 Feb 14;22:101356. doi: 10.1016/j.onehlt.2026.101356 (PMC12955692; doi:10.1016/j.onehlt.2026.101356)
Supplement: Supplementary file 1 — Supplementary material [file mmc1.pdf]

SUPPLEMENTARY INFORMATION

Table S1. Data of training and testing dataset for the *Mycobacterium tuberculosis* complex classifiers.

Species	Isolate ID	Number of FT-IR spectra	Training Dataset	Testing dataset
M. bovis	309	20		X
	779	16	X	
	B2	14		X
	Cap	13	X	
	Lh1	17		X
	Lh2	16		X
	SP38	17	X	
M. tuberculosis	11/21P	15	X	
	12/24A	15	X	
	CDC1551	18		X
	H37Rv	36		X
	M299	15		X
	SVOC1	16	X	
	SVO68	14	X	
Total		242	124	118

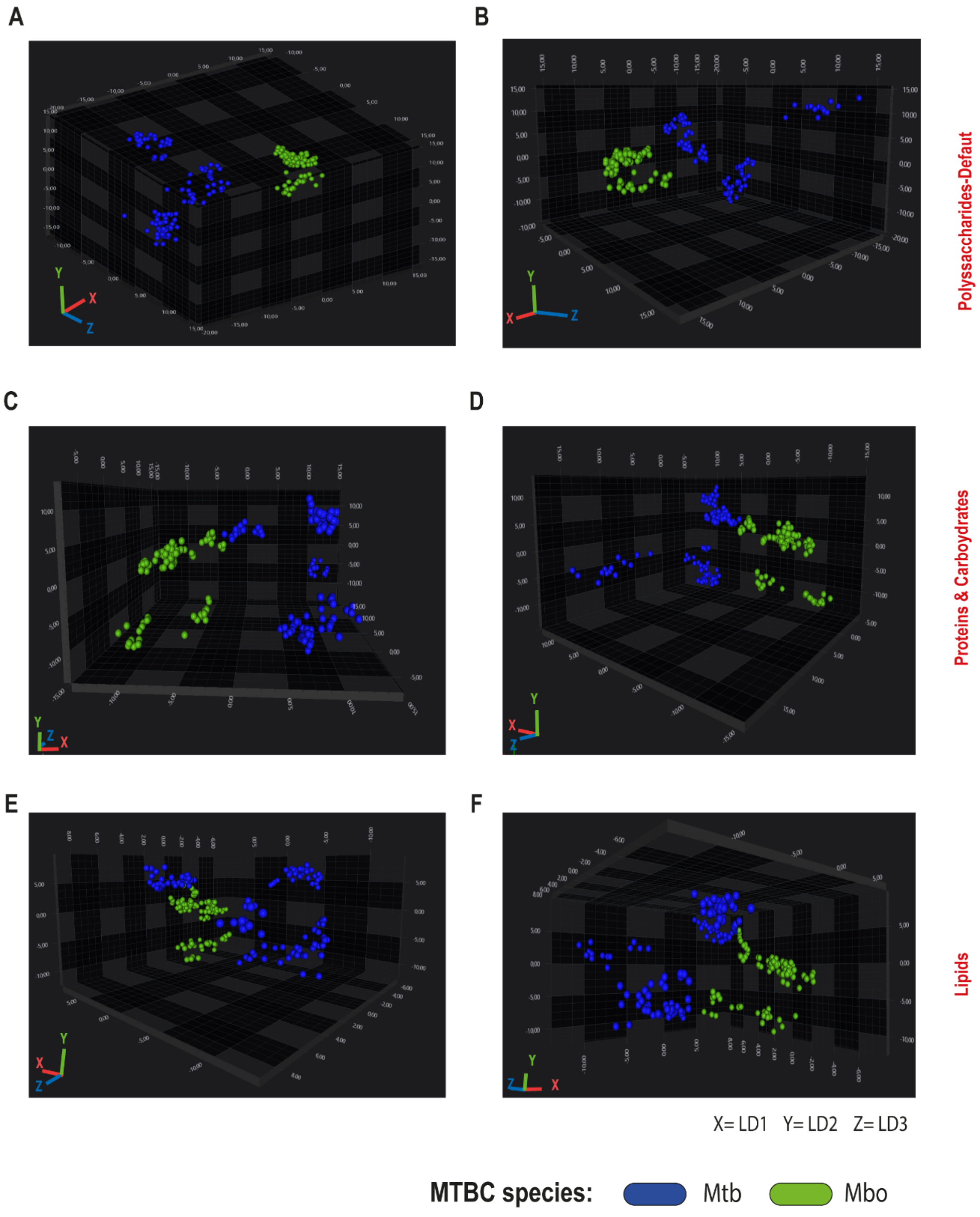

Figure S1. Linear Discriminant Analysis (LDA) of isolates of *Mycobacterium tuberculosis* (Mtb, blue) and *Mycobacterium bovis* (Mbo, green) subjected to Fourier Transform Infrared Spectroscopy. (A/B) 3D scatter plot represented at different perspectives of polysaccharide-based spectra of Mtb and Mbo strains (LDA: 30 LDs, 99.1% variance, target group = isolate ID). The x-axis shows LD1 (59.00%) the y-axis shows LD2 (15.78%) and z-axis shows LD3 (10.76%), together displaying 85.54% of the variance. **(C/D)** 3D scatter plot represented at different perspectives of protein/carbohydrate-based spectra of Mtb and Mbo strains (LDA: 30 LDs, 99.7% variance, target group =

isolate ID). The x-axis shows LD1 (41.29%) the y-axis shows LD2 (32.66%) and z-axis shows LD3 (9.91%), together displaying 83.87% of the variance. **(E/F)** 3D scatter plot represented at different perspectives of lipid-based spectra of Mtb and Mbo strains (LDA: 30 LDs, 100% variance, target group = isolate ID). The x-axis shows LD1 (48.13%) the y-axis shows LD2 (31.58%) and z-axis shows LD3 (10.12%), together displaying 89.83% of the variance.

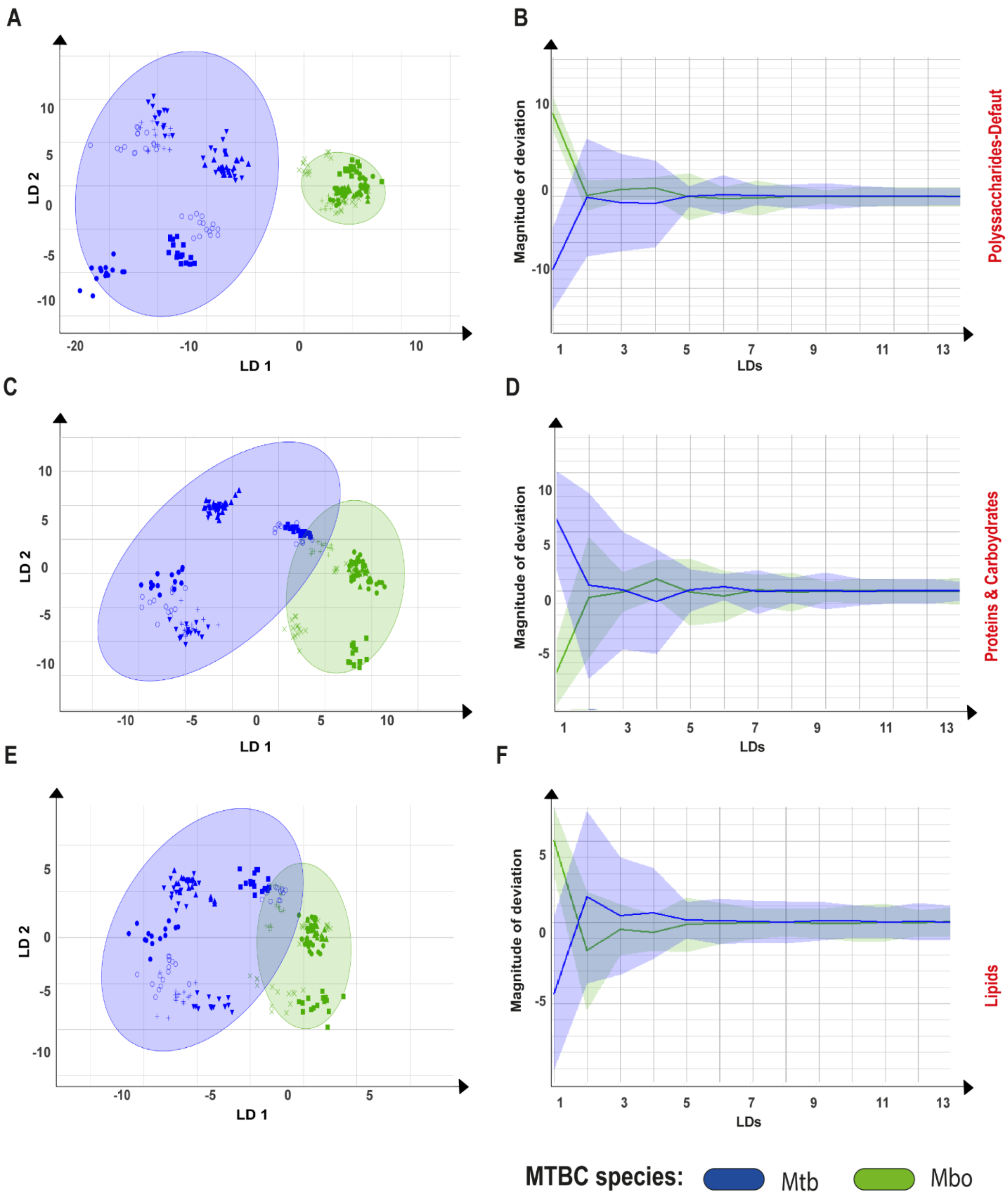

A
B
C
D
E
F
LD 1
LD 2
LDs
Magnitude of deviation
Polyssaccharides-Defaut
Proteins & Carboydrates
Lipids
MTBC species:
Mtb
Mbo

Figure S2. Linear Discriminant Analysis (LDA) and deviation plots of isolates of *Mycobacterium tuberculosis* (Mtb, blue) and *Mycobacterium bovis* (Mbo, green) subjected to Fourier Transform Infrared Spectroscopy. In the LDA plots, isolates are represented by different shapes and are encircled to indicate their replicates. **(A)** 2D scatter plot of polysaccharide-based spectra of Mtb and Mbo strains (LDA: 30 LDs, 99.1% variance, target group = isolate ID). The x-axis shows LD1 (59%) and the y-axis shows LD2 (15.78%), together displaying 74.78% of the variance. **(B)** Deviation plot of polysaccharide-based spectra of Mtb and Mbo strains. The solid line indicates the mean spectrum of all isolates, and the shaded area represents the standard deviation. The x-axis displays different linear components. **(C)** 2D scatter plot of protein/carbohydrate-based spectra of Mtb and Mbo strains (LDA: 30 LDs, 99.7% variance, target group = isolate ID). The x-axis shows LD1 (41.29%) and the y-axis shows LD2 (32.66%), together displaying 73.95% of the variance. **(D)** Deviation plot of protein/carbohydrate-based spectra of Mtb and Mbo strains. The solid line indicates the mean spectrum, and the shaded area represents the standard deviation. The x-axis displays different principal components. **(E)** 2D scatter plot of lipid-based spectra of Mtb and Mbo strains (LDA: 30 LDs, 100% variance, target group = isolate ID). The x-axis shows LD1 (48.13%) and the y-axis shows LD2 (31.58%), together displaying 79.71% of the variance. **(F)** Deviation plot of lipid-based spectra of Mtb and Mbo strains. The solid line indicates the mean spectrum, and the shaded area represents the standard deviation. The x-axis displays different principal components.

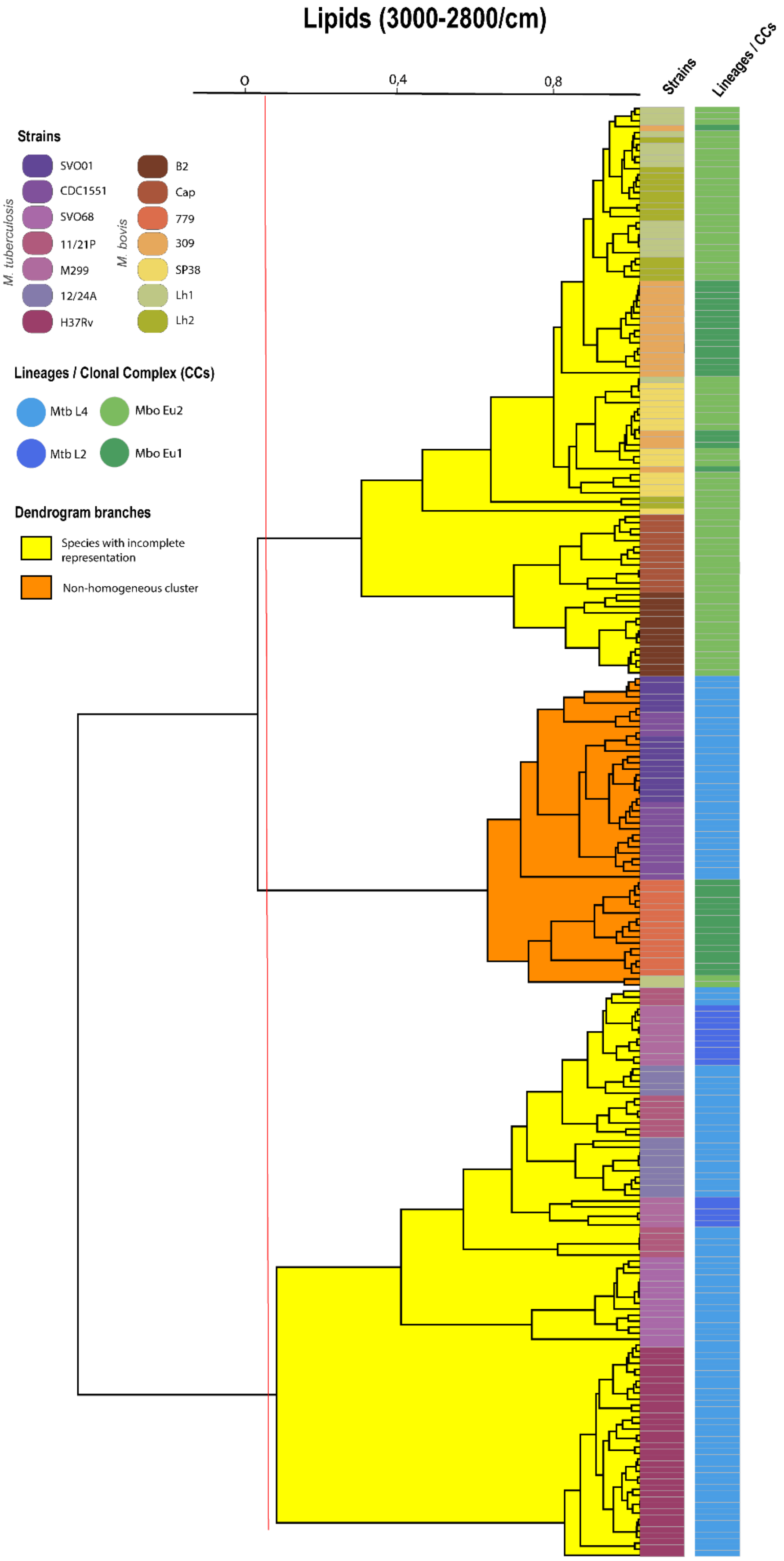

Lipids (3000-2800/cm)
0
0,4
0,8
Strains
Lineages / CCs
Strains
M. tuberculosis
SVO01
CDC1551
SVO68
11/21P
M299
12/24A
H37Rv
M. bovis
B2
Cap
779
309
SP38
Lh1
Lh2
Lineages / Clonal Complex (CCs)
Mtb L4
Mbo Eu2
Mtb L2
Mbo Eu1
Dendrogram branches
Species with incomplete representation
Non-homogeneous cluster

Figure S3. Dendrogram based on the lipid spectral region of *Mycobacterium tuberculosis* (Mtb) and *Mycobacterium bovis* (Mbo) isolates analyzed by Fourier Transform Infrared Spectroscopy (FT-IRS). Spectral splicing was performed using the lipid range (3000–2800 cm^{-1}). The UPGMA algorithm with correlation distance was applied. The horizontal axis represents spectral distance, indicating differences between FT-IRS spectra of the isolates. Spectral distance cutoff, automatically determined by the software using the Spectral Distance Index (SDI) and mean Concordance (mC), is shown as a vertical line intersecting the dendrogram branches. This cutoff highlights two-homogeneous (one species only, yellow-colored branches) and one non-homogenous cluster (two species, orange-colored branches) composed of Mtb and Mbo strains.

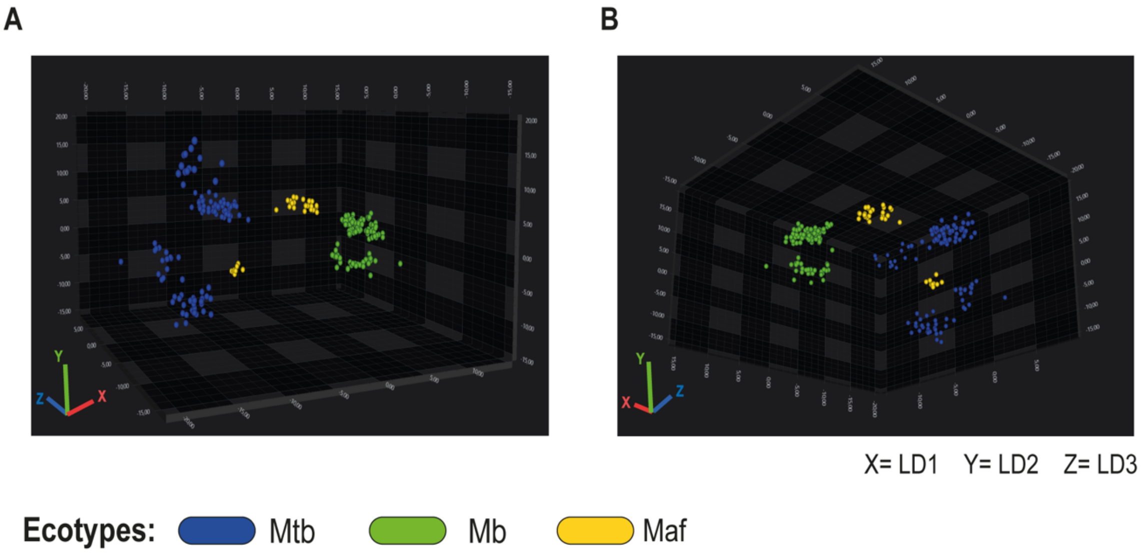

Figure S4. Linear Discriminant Analysis (LDA) of *Mycobacterium tuberculosis* (Mtb, blue), *Mycobacterium bovis* (Mbo, green), and *Mycobacterium africanum* (Maf, yellow) isolates subjected to Fourier Transform Infrared Spectroscopy. 3D scatter plots (LDA 30 LDs, 99.3% variance, target group = isolate ID) showing Mtb isolates in blue, Maf in yellow and Mbo in green. A and B show different perspectives of the same scatter plot. The x-axis shows LD1 (54.97%), y-axis shows LD2 (19.40%), z-axis shows LD3 (8.45%), together displaying 82,82% of the variance.

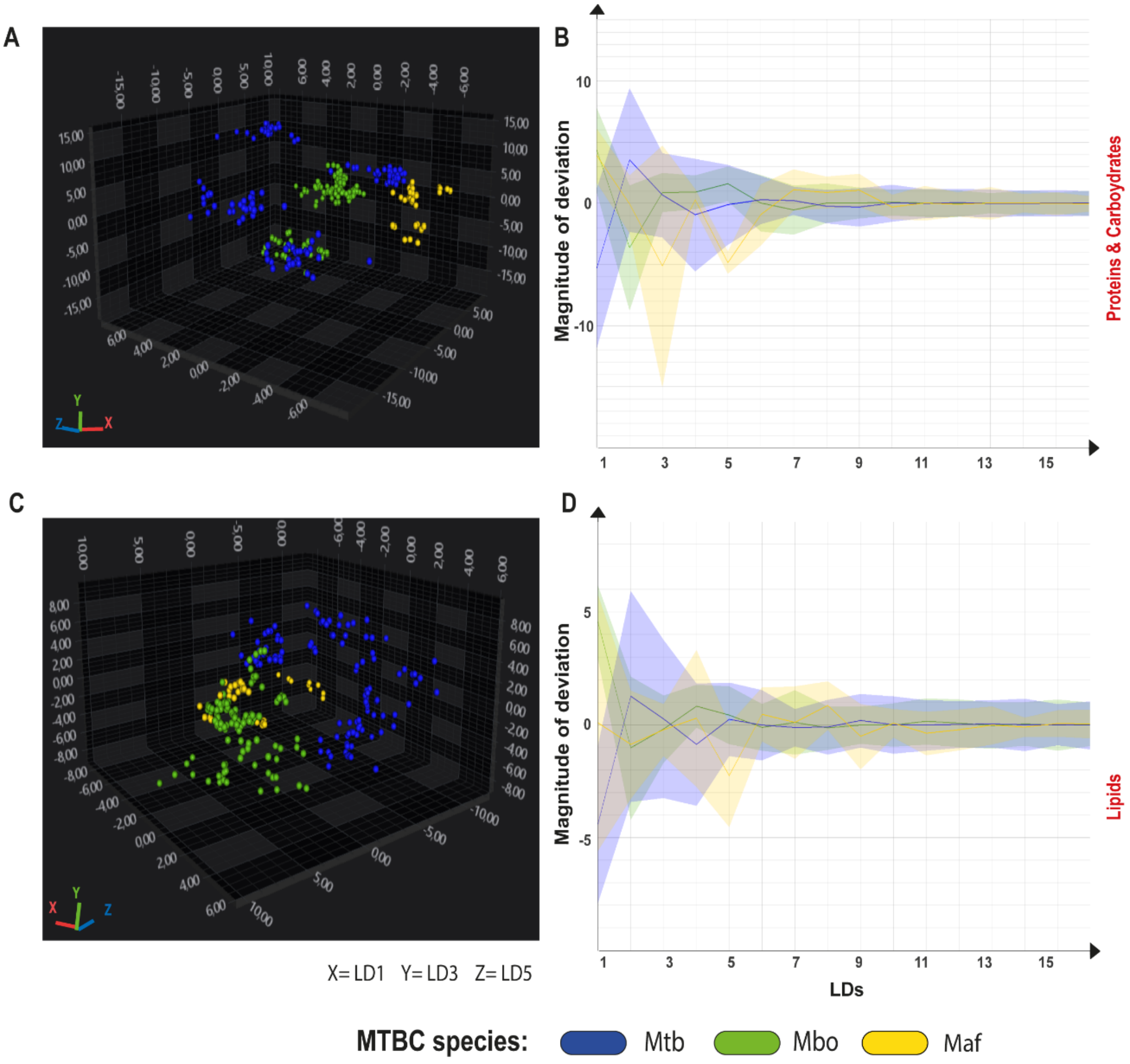

Figure S5. Linear Discriminant Analysis (LDA) and deviation plots of isolates of *Mycobacterium tuberculosis* (Mtb, blue), *Mycobacterium bovis* (Mbo, green) and *Mycobacterium africanum* (Maf, yellow) subjected to Fourier Transform Infrared Spectroscopy. **(A)** 3D scatter plot of protein/carbohydrate-based spectra of Mtb, Mbo and Maf strains (LDA: 30 LDs, 99.7% variance, target group = isolate ID). The x-axis shows LD1 (34.29%), y-axis shows LD3 (16.26%) and z-axis shows LD5 (6.58%), together displaying 57.13% of the variance. **(B)** Deviation plot of protein/carbohydrate-based spectra of Mtb, Mbo and Maf strains. The solid line indicates the mean spectrum, and the shaded area represents the standard deviation. The x-axis displays different linear components. **(C)** 3D scatter plot lipid-based spectra of Mtb, Mbo and Maf strains (LDA: 30 LDs, 100% variance, target group = isolate ID). The x-axis shows LD1 (47.15%), y-axis shows LD3 (9.82%) and z-axis shows LD5 (4.13%), together displaying 61.1% of the variance. **(D)** Deviation plot of lipid-based spectra of Mtb, Mbo and Maf strains. The solid line indicates the mean spectrum, and the shaded area represents the standard deviation. The x-axis displays different linear components.
